# Association of the epithelial-to-mesenchymal transition phenotype with responsiveness to the p21-activated kinase inhibitor, PF-3758309, in colon cancer models

**DOI:** 10.3389/fphar.2013.00035

**Published:** 2013-03-28

**Authors:** Todd M. Pitts, Gillian N. Kulikowski, Aik-Choon Tan, Brion W. Murray, John J. Arcaroli, John J. Tentler, Anna Spreafico, Heather M. Selby, Maria I. Kachaeva, Kelly L. McPhillips, Blair C. Britt, Erica L. Bradshaw-Pierce, Wells A. Messersmith, Marileila Varella-Garcia, S. Gail Eckhardt

**Affiliations:** ^1^Division of Medical Oncology, University of Colorado Anschutz Medical CampusAurora, CO, USA; ^2^Pfizer Global Research and Development, La Jolla LaboratoriesSan Diego, CA, USA; ^3^University of Colorado Cancer Center, University of Colorado Anschutz Medical CampusAurora, CO, USA

**Keywords:** PAK, PF-3758309, colorectal cancer, intrinsic resistance, EMT

## Abstract

The p21-activated kinase (PAK) family of serine/threonine kinases, which are overexpressed in several cancer types, are critical mediators of cell survival, motility, mitosis, transcription, and translation. In the study presented here, we utilized a panel of colorectal cancer (CRC) cell lines to identify potential biomarkers of sensitivity or resistance that may be used to individualize therapy to the PAK inhibitor PF-03758309. We observed a wide range of proliferative responses in the CRC cell lines exposed to PF-03758309, this response was recapitulated in other phenotypic assays such as anchorage-independent growth, three-dimensional (3D) tumor spheroid formation, and migration. Interestingly, we observed that cells most sensitive to PF-03758309 exhibited up-regulation of genes associated with a mesenchymal phenotype (CALD1, VIM, ZEB1) and cells more resistant had an up-regulation of genes associated with an epithelial phenotype (CLDN2, CDH1, CLDN3, CDH17) allowing us to derive an epithelial-to-mesenchymal transition (EMT) gene signature for this agent. We assessed the functional role of EMT-associated genes in mediating responsiveness to PF-3758309, by targeting known genes and transcriptional regulators of EMT. We observed that suppression of genes associated with the mesenchymal phenotype conferred resistance to PF-3758309, *in vitro* and *in vivo*. These results indicate that PAK inhibition is associated with a unique response phenotype in CRC and that further studies should be conducted to facilitate both patient selection and rational combination strategies with these agents.

## INTRODUCTION

Substantial progress has been made over the last decade in the development of effective therapy for patients diagnosed with colorectal cancer (CRC) due to the advent of targeted therapies (Siddiqui and Piperdi, 2010); however, CRC remains the third leading cause of both cancer incidence and death (Jemal et al., 2011). Currently, the approved agents for treatment of CRC include monoclonal antibodies or small molecule tyrosine kinase inhibitors against the epidermal growth factor receptor (EGFR) and vascular endothelial growth factor (VEGF) pathways, as well as the standard chemotherapeutic agents 5-FU, irinotecan, and oxaliplatin (Ortega et al., 2010; Bagnasco et al., 2012; Strumberg et al., 2012). Current research in this disease is primarily focused on various iterations of these therapies, while novel agent development is stalled. Despite the advances made in the treatment of CRC, agents that target new pathways need to be explored in concert with strategies that identify resistance mechanisms and incorporate biomarkers that can enhance patient selection and thus, clinical benefit.

An emerging target for cancer therapy is the p21-activated kinase (PAK) family of proteins that appear promising due to the alterations of PAKs detected in a wide variety of human malignancies (Kumar et al., 2006; Eswaran et al., 2009; Molli et al., 2009). The PAKs are important effectors of the Rho family GTPases that regulate many intracellular functions and act as molecular switches regulating diverse cellular processes (Wells et al., 2005). There are six PAK isoforms, and based upon structural or functional similarities, belong to group I (PAK1–3) or group II (PAK4–6; Murray et al., 2010). Group I PAKs are activated by extracellular signals in both GTPase-dependent and -independent mechanisms, whereas, Group II PAKs are constitutively active (Molli et al., 2009). The PAKs play an essential role in cell signaling and control cellular functions such as proliferation, survival, angiogenesis, mitosis, transcription, and translation (Kumar et al., 2006; Eswaran et al., 2009; Molli et al., 2009). PAKs also contribute to the regulation of growth factor signaling, reorganization of the cytoskeleton, and growth factor-mediated migration (Eswaran et al., 2009; Molli et al., 2009). PAKs are often up-regulated or hyperactive in a variety of human cancers (Kumar et al., 2006). The overexpression of PAKs in cancer has been linked to increased migratory potential, anchorage-independent growth, oncogenic transformation, and metastasis (Eswaran et al., 2009; Murray et al., 2010), thereby leading to a more aggressive phenotype. The involvement of the different isoforms of PAK family members in cancer provides a rationale for the development of agents targeting these proteins (Kumar et al., 2006).

PF-3758309 is a potent ATP-competitive pyrrolopyrazole inhibitor of PAK1 and PAK4 with antitumor activity against a broad range of tumor types (Murray et al., 2010). PAK1 is thought to play a role in the human epidermal growth factor receptor 2 (HER-2)-mediated transformation of breast caner cells in which it cooperates with other oncogenes in transforming epithelial cells (Arias-Romero et al., 2010). Other studies indicate that PAK1 is activated by Akt resulting in phosphorylation of Bad and inhibition of apoptosis (Schurmann et al., 2000; Tang et al., 2000). Thus, inhibition of PAK1 reduces Bad phosphorylation and may be a strategy to induce apoptosis (Deacon et al., 2003). Another target of this agent, PAK4, while required for normal development, is also the only PAK member that is oncogenic when overexpressed (Wells and Jones, 2010). PAK4 overexpression is observed at a high frequency in several malignancies including those of colon, breast, pancreatic, thyroid, and ovarian origin (Callow et al., 2002; Liu et al., 2008; Molli et al., 2009). PAK4 has been shown to promote cell survival through the phosphorylation and subsequent inactivation of the pro-apoptotic factor BAD (Bcl-2-associated death promoter), through initiation of tumor necrosis factor-alpha (TNF-α)-stimulated survival pathways, and by inhibiting caspase-3 activation (Gnesutta et al., 2001; Eswaran et al., 2009). In addition to promoting cell survival, PAK4 is capable of inducing morphological changes through the loss of stress fibers and focal adhesions, decreased spreading, and cell rounding (Qu et al., 2001; Wells and Jones, 2010). This ability to modulate the actin cytoskeleton results from both kinase-dependent and -independent mechanisms, although kinase activity is required for Ras-driven anchorage-independent growth, supporting the role of a PAK4 selective inhibitor (Murray et al., 2010). However, this has been challenging due to the functional similarities of the PAK family members, the multiple signaling pathways that activate PAKs, and the fact that some of the cancer promoting functions are not dependent on its kinase domain (Kumar et al., 2006; Molli et al., 2009).

The development of predictive biomarkers is becoming increasingly critical to successful early drug development. One example very relevant to CRC, is the finding that mutation of the KRAS (V-Ki-ras2 Kirsten rat sarcoma viral oncogene homolog) gene is a strong negative predictor for responsiveness of patients to the EGFR-targeted agents, cetuximab and panitumumab (Banck and Grothey, 2009; Prenen et al., 2010; Siddiqui and Piperdi, 2010). More recent examples include the positive predictive markers of BRAF (v-raf murine sarcoma viral oncogene homolog B1) mutation and ALK (anaplastic lymphoma kinase) gene arrangement, both of which have been applied in the early clinical development of the BRAF kinase inhibitor, PLX4032, and the ALK inhibitor, crizotinib, respectively (Heakal et al., 2011; Shaw et al., 2011). Thus, the goal of this study was to not only assess the efficacy of the PAK inhibitor, PF-03758309, against a panel of CRC cell lines, but to also identify potential biomarkers of responsiveness that could be applied in early clinical development. In conducting these studies, we identified several core genes associated with the epithelial-to-mesenchymal transition (EMT) phenotype as predictive for responsiveness to PF-03758309, and thus focused studies on assessing the functional relationship between EMT and PF-03758309, as well as the potential of a rational combination strategy.

## MATERIALS AND METHODS

### CELL CULTURE AND PROLIFERATION

Twenty-seven human colon cancer cell lines were obtained from American Type Culture Collection (ATCC, Manassas, VA, USA). The GEO cells were a generous gift from Dr. Fortunato Ciardiello (Cattedra di Oncologia Medica, Dipartimento Medico-Chirurgico di Internistica Clinica e Sperimentale “F Magrassi e A Lanzara,” Seconda Università degli Studi di Napoli, Naples). All cells except GEO were grown in Roswell Park Memorial Institute (RPMI) medium supplemented with 10% fetal bovine serum, 1% non-essential amino acids, 1% penicillin/streptomycin and were maintained at 37°C in an incubator under an atmosphere containing 5% CO_2_. GEO cells were grown in Dulbecco’s modified Eagle’s medium (DMEM)/F12 supplemented with 10% fetal bovine serum, 1% non-essential amino acids, and 1% penicillin/streptomycin. The cells were routinely screened for the presence of mycoplasma (MycoAlert, Cambrex Bio Science, Baltimore, MD, USA) and were exposed to PF-3758309 when they reached approximately 70% confluence. All cell lines were tested and authenticated in the University of Colorado Cancer Center DNA Sequencing and Analysis Core. CRC cell line DNA was tested using the Profiler Plus Kit (Applied Biosystems, Foster City, CA, USA) and compared to that from the ATCC. PF-3758309 was provided by Pfizer, Inc. (San Diego, CA, USA) and prepared as a 10-mM stock solution in dimethyl sulfoxide (DMSO). Cytotoxic/proliferation effects were determined using the sulforhodamine B (SRB) method (Skehan et al., 1990). Briefly, cells in logarithmic growth phase were transferred to 96-well flat bottom plates with lids. One hundred microliters of cell suspensions containing 1,500–5,000 viable cells were plated into each well and incubated overnight prior to exposure with increasing concentrations of PF-3758309 for 72 h. Media was removed and cells were fixed with cold 10% trichloroacetic acid for 30 min at 4°C. Cells were then washed with water and stained with 0.4% SRB (Fisher Scientific, Pittsburgh, PA, USA) for 30 min at room temperature, washed again with 1% acetic acid, followed by stain solubilization with 10 mM Tris at room temperature. The plate was then read on a plate reader (Biotek Synergy 2, Winooski, VT, USA) set at an absorbance wavelength of 565 nm. Cell proliferation curves were derived from the raw absorbance (optical density, OD) data.

### FLUORESCENCE *IN SITU* HYBRIDIZATION

Dual-color fluorescence in situ hybridization (FISH) assay was performed according to standard protocol in the laboratory. Briefly, the slides were first washed in 70% acetic acid for 5–40 s, then incubated in 0.008% pepsin/0.01 M HCl at 37°C for 3–6 min, in 1% formaldehyde for 8 min and dehydrated in a graded ethanol series. The probe mix consisting of 50–100 ng of PAK4 and 100–150 ng of CCNE1 per 113 mm^2^ area was applied to the selected hybridization areas, which were covered with glass coverslips and sealed with rubber cement. DNA co-denaturation was performed for 7 min at 85°C and hybridization was allowed to occur at 37°C for 16–21 h. Post-hybridization washes were performed with 2× SSC/0.3% NP-40 at 72°C and 2× SSC for 2 min at room temperature and specimens were then dehydrated in a graded ethanol series. Chromatin was counterstained with 4′,6-diamidino-2-phenylindole (DAPI; 0.3 μg/ml in Vectashield Mounting Medium, Vector Laboratories).

Analysis was performed on epifluorescence microscope using single interference filter sets for green (fluorescein isothiocyanate, FITC), red (Texas Red), and blue (DAPI) as well as dual (red/green) and triple (blue, red, green) band pass filters. At least 20 metaphase spreads and 100 interphase nuclei were analyzed in each cell line. Images were captured using the CytoVision software (Applied Imaging Inc., San Jose, CA, USA).

### SNP-ARRAY ANALYSIS

DNA was extracted using the Quick g-DNA Mini kit (Zymo Research, Irvine, CA, USA) according to the manufacturer’s instructions. Genome-Wide Human SNP Array 6.0 (Affymetrix, CA, USA) was used for copy number variation analysis as per the manufacturer’s instructions. Raw data from the SNP Array 6.0 were normalized and extracted using Partek Genomics Software Suite. Copy number analysis was performed using the segmentation algorithm contains in the copy number workflow.

### KRAS MUTATION ANALYSIS

DNA was isolated from CRC cell lines using the Quick g-DNA Mini kit (Zymo Research, Irvine, CA, USA) using the manufactures instructions. Exon 2 of the KRAS gene was amplified, as described previously (Pitts et al., 2010) and the polymerase chain reaction (PCR) product was sequenced in the University of Colorado Cancer Center DNA Sequencing and Analysis Core.

### APOPTOSIS

Apoptosis was measured using a Caspase-Glo 3/7 assay (Promega Corporation, Madison, WI, USA). One hundred microliters of cell suspensions containing 1,500–5,000 cells were plated in a 96-well, white-walled tissue culture plate and allowed to adhere overnight. Media was removed and 100 μL of media with and without PF-3758309 was added and cells were exposed for 24, 48, and 72 h. Following exposure to PF-3758309, 100 μL of Promega Glo Caspase 3/7 reagent was added, cells were incubated in the dark for 1 h, and the plate was read on a plate reader (Biotek Synergy 2, Winooski, VT, USA) using luminescence as the determinant of apoptosis. An increase in luminescence indicated an increase in apoptosis; the data was then normalized to the untreated cells.

### HUMAN PHOSPHO-KINASE ARRAY

Cells were plated in six-well plates and incubated overnight. Following a 24-h exposure to PF-3758309 (1.0 μmol/L), cells were rinsed once with phosphate-buffered saline (PBS) and solubilized with lysis buffer. Lysates were gently rocked at 4°C for 30 min, then microcentrifuged for 5 min at 14,000 × *g*. Supernatants were transferred to clean microcentrifuge tubes and total protein was quantified (as previously described). Two hundred micrograms of lysates were diluted and incubated with the Human Phospho-Kinase Proteome Profiler Array (R&D Systems) according to the manufacturer’s protocol. Membranes were then scanned and density was measured.

### GENE EXPRESSION PROFILES

Cells were plated at 2 × 10^6^ in six-well plates 24 h prior to harvest. After 24–72 h cells were rinsed twice with PBS, and RNA was prepared using a RNeasy Plus mini kit (Qiagen, Valencia, CA, USA). RNA stabilization, isolation, and microarray sample labeling were carried out using standard methods for reverse transcription (RT) and one round of *in vitro* transcription. Total RNA isolated from CRC cell lines and tumor xenografts was hybridized on Affymetrix U133 Plus 2.0 gene arrays at least in duplicates. The sample preparation and processing procedure was performed as described in the Affymetrix GeneChip® Expression Analysis Manual (Affymetrix Inc., Santa Clara, CA, USA). In addition, CRC cell line gene expression profiles were obtained from the GlaxoSmithKline (GSK) genomic profiling data via the National Cancer Institute (NCI) cancer Bioinformatics Grid (caBIG®) website ^1^. These data were also profiled using Affymetrix U133 Plus 2.0 gene arrays in triplicates. To integrate the data generated from our lab and GSK, absolute intensity signals from the microarray gene expression profiles were extracted and probe sets representing the same gene were collapsed based on maximum values. Next, the gene expression levels were converted to a rank-based matrix and standardized (mean = 0, SD = 1) for each microarray. Using this pre-processing method, the same cell lines from different data sets were clustered based on their gene expression profiles. Data analyses were performed on this rank-based matrix. Raw expression data is available online ^2^.

### SEMI-QUANTITATIVE RT-PCR FOR EMT MARKERS

Total RNA was isolated from cells using the RNeasy mini kit (Qiagen, Valencia, CA, USA), cDNA synthesized from 1 μg of total RNA using the Verso cDNA Kit (ABgene, Surrey, KT, UK), and expression levels detected from 100 ng of cDNA using Solaris qPCR Gene Expression Assays (Dharmacon, Lafayette, CO, USA). The primer/probes used were vimentin, caldesmon, Zeb1, and E-cadherin.

### ANCHORAGE-INDEPENDENT GROWTH AND THREE-DIMENSIONAL ASSAYS

A soft agar colony-forming assay was conducted to determine the effects of PF-3758309 on anchorage-independent growth. Cells were seeded in a six-well plate on top of a 0.6% agar bed at a density of 10^4^ cells per well in 0.4% agar. After 4 days, 15 nmol/L PF-3758309 was added and replenished twice weekly for 2 weeks. Upon completion, wells were stained with 1 mL nitro blue tetrazolium and photographed at 4× magnification. Three-dimensional culture was performed using eight-chamber polystyrene vessel glass slides (BD Falcon, Franklin Lakes, NJ, USA). Cells were seeded at a density of 2,500 cells/well in a 2% matrigel/media mixture and were added to the top of a solidified layer of matrigel in the glass slides. Spheroids were allowed to form for 4 days prior to the addition of PF-3758309. Media was replaced twice weekly for 2 weeks and photographed at 4× magnification.

For both assays the speroid/colonies were counted and graphs generated using the Graph Pad Program (La Jolla, CA, USA)

### MIGRATION ASSAYS

Cell migration was measured using a wound healing assay (scratch) and a modified Boyden chamber. For the scratch assay, cells were plated in six-well plates under standard growth conditions. Once the cells reached 100% confluency, a scratch was made by moving a sterile pipette tip along the bottom of the well. Cells were then incubated in the presence and absence of 15 nM PF-3758309 for 48 h. Following exposure, cells were fixed with 90% methanol for 5 min, stained with 0.5% crystal violet for 20 min, and washed with water. Photographs were then taken at 20× magnification on an inverted microscope for a qualitative assessment of migratory inhibition upon treatment with PF-3758309.

### *IN VIVO* XENOGRAFT TREATMENT

Five- to six-week-old female athymic nude mice (Harlan Sprague Dawley) were used. Mice were caged in five groups and kept on a 12-h light/dark cycle and provided with sterilized food and water ad libitum. Animals were allowed to acclimate for at least 7 days before any handling. All CRC cells were harvested in exponential phase growth and resuspended in a 1:1 mixture of serum-free RPMI 1640 and Matrigel (BD Biosciences). Five to ten million cells per mouse were injected s.c. into the flank using a 23-gage needle. Mice were monitored daily for signs of toxicity and were weighed twice weekly. Tumor size was evaluated twice per week by caliper measurements using the following formula: tumor volume = length × width^2^ × 0.52. When tumors reached 150–300 mm^3^ mice were randomized into two groups with at least 10 tumors per group. Mice were then treated for 14 days with either vehicle control (0.5% methylcellulose), or PF-3758309 (25 mg/kg) twice daily by oral gavage. All of the xenograft studies were conducted in accordance with the National Institutes of Health (NIH) guidelines for the care and use of laboratory animals were conducted in a facility accredited by the American Association for Accreditation of Laboratory Animal Care, and received approval from University of Colorado Animal Care and Use Committee prior to initiation.

### shRNA KNOCKDOWN/miRNA EXPRESSION

The pRS–shE2F6 gene-specific short hairpin RNA (shRNA) expression cassettes, along with control shRNA plasmids including the original pRS vector (TR20003, were purchased from OriGene (Rockville, MD, USA). The sequence of the E-cadherin 29-mer shRNA is GCTACAGA-CAATGGTTCTCCAGTTGCTAC, vimentin-ACTTCTCAGCATCACGATGACCTTGAATA, caldesmon-AAGAATCGCCTACC-AGAGGAATGACGATG, and Zeb-1-TAGGCGAGAGTAGTGAGCAAGTGTCT-GAA. The sensitive cell lines were also transfected with a plasmid containing microRNA (miRNA) 200c (kindly provided by Jennifer Richer, University of Colorado Anschutz Medical Campus). Stable clones were generated by transfecting the PF-3758309 resistant (HT29) and sensitive (RKO, HCT116, SW620) cells in six-well dishes with 1 μg of each of the shRNA plasmids using Fugene 6 (Roche, Basel, Switzerland), according to the manufacturer’s recommendations. Seventy-two hours after transfection, the cells were placed under selection with 2.5 μg/mL of puromycin, splitting 1:5 when the cells reached confluency. Multiple clones from the same transfection were pooled and grown under puromycin selection. Successful knockdown of specific genes and gene products was confirmed by semi-quantitative RT-PCR and immunoblotting with specific antibodies. Each experiment was conducted in triplicate. Following selection and confirmation of knockdown or expression the cell lines were tested for proliferation or in 3D culture as described above.

### STATISTICAL METHODS

Statistical analysis was performed using GraphPad Prism Software (La Jolla, CA, USA). For comparisons of two groups an un-paired *t*-test was performed. When comparisons of multiple groups were needed an analysis of variance (ANOVA) was performed. The specific tests applied are included in the figure legends.

## RESULTS

### ASSESSMENT OF RESPONSIVENESS OF A PANEL OF CRC CELL LINES TO PF-3758309

**FIGURE 1:**
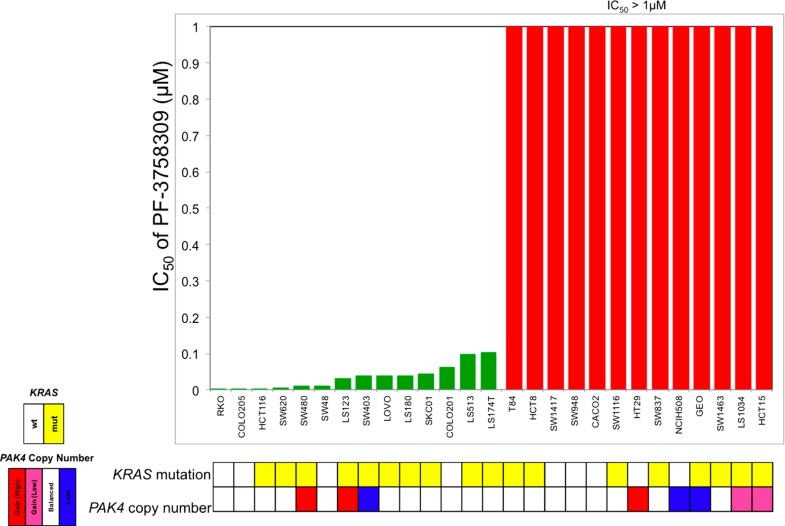
**Effect of PF-3758309 on proliferation after 72 h in a panel of 27 colorectal cancer cell lines.** Absolute IC_50_ values were calculated and graphed (green, sensitive; red, resistant). KRAS mutational status was obtained by direct sequencing and PAK4 gene amplification was obtained by fluorescence *in situ* hybridization.

**FIGURE 2:**
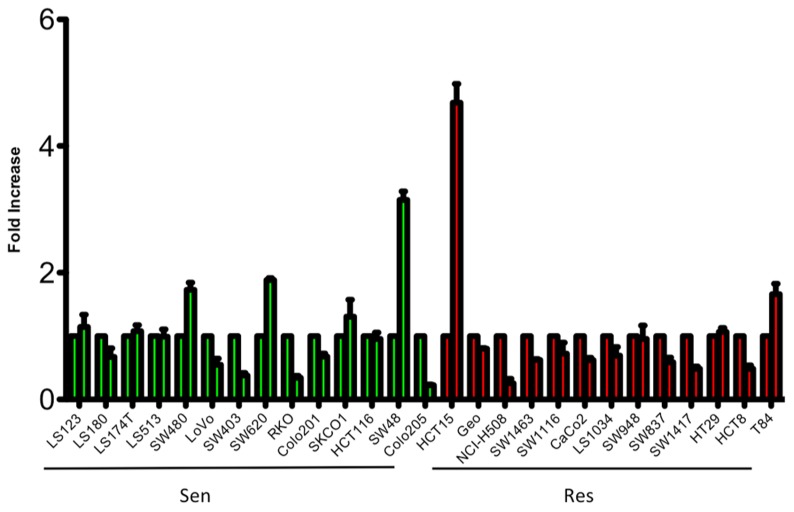
**Induction of apoptosis of CRC cell lines following 72-h treatment with PF-3758309 at concentration 1.0 μmol/L**.

To evaluate the sensitivity of CRC cell lines to PF-3758309, a panel of 27 CRC cell lines were exposed to increasing concentrations and assessed for proliferation using the SRB assay. As depicted in **Figure 1**, there was a broad range of sensitivity of the CRC cell lines to PF-3758309. For categorization, a sensitive cell line was classified as one with an IC_50_ <0.0125 μmol/L, whereas resistant cell lines had IC_50_ values ≥ 1 μmol/L; 14 cell lines met the criteria as being sensitive, and the remaining 13 were resistant. We did not identify a relationship between responsiveness to PF-3758309 and either the presence of KRAS mutation or increased copy number of PAK4 by FISH (**Figure 1**). Copy number of the six PAK isoforms was analyzed using SNP-array analysis. No amplification of any of the PAKs was observed. In addition to proliferation, apoptosis was evaluated using a caspase 3/7 assay. Apoptosis was observed at 24, 48, and 72 h post exposure in 9 of the 27 cell lines, with the greatest increase seen at 72 h (**Figure 2**). There was no significant correlation between cell lines deemed sensitive in regards to proliferation and those that demonstrated an increase in apoptosis upon treatment with PF-3758309 (*p* = 0.23).

### EVALUATION OF DOWNSTREAM EFFECTORS BY A PHOSPHO-KINASE ARRAY AND IMMUNOBLOTTING

**FIGURE 3:**
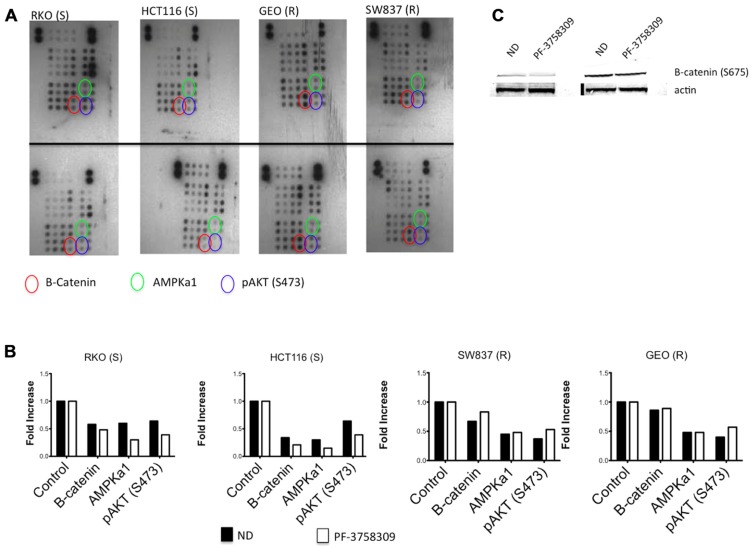
**Proteome profiler analysis of two sensitive and two resistant CRC cell lines to PF-3758309**. **(A)** Total protein lysates from control (top) and PF-3758309 treated (bottom) were analyzed by a human phospho-kinase array. **(B)** Semi-quantitative analysis of the spots was measured by densitometry and the mean (*n* = 2 spots) is presented in the graphs as a fold increase over control. **(C)** HCT116 and GEO were treated with 1 μmol/L of PF-3758309 for 8 h and levels of B-catenin were assessed.

To assess the activity of PF-3758309 on a spectrum of kinases, we evaluated its effects on two sensitive and two resistant CRC cell lines using a proteome array with 46 different kinase phosphorylation sites. The proteome array demonstrated that regardless of sensitivity, members of the Src kinases, pERK and pMEK were inhibited following exposure to PF-3758309 (data not shown). However, when comparing the sensitive versus resistant CRC cell lines, we observed that β-catenin, AMPKα1, and AKT (S473) were all suppressed in the sensitive lines following exposure to PF-3758309 (**Figure 3**). Since recent reports suggest that PAKs can directly phosphorylate β-catenin at S675 to stabilize it we decided to see if that was the case in CRC. Indeed we observed a decrease in P-S675 in the PF-3758309 sensitive cell line, HCT116 and not in the resistant GEO cell line (**Figure 3**).

### IDENTIFICATION OF DIFFERENTIALLY EXPRESSED GENES BETWEEN CRC CELL LINES SENSITIVE OR RESISTANT TO PF-3758309

**FIGURE 4:**
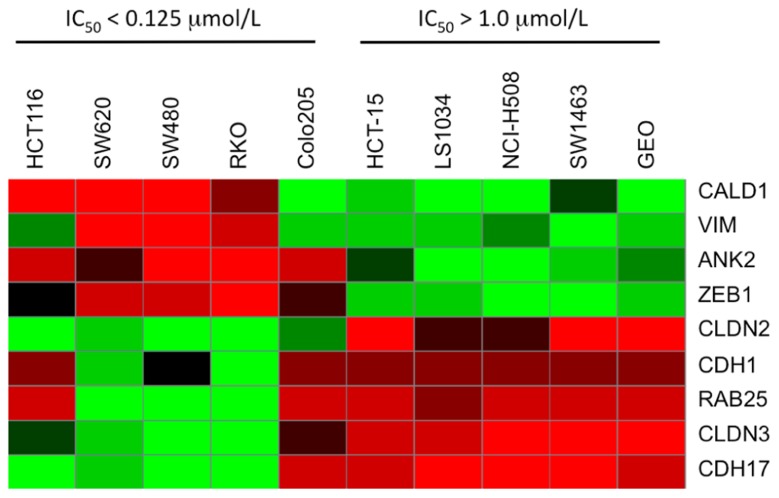
**Heat map showing differential expression of selected genes involved in EMT compared to sensitivity to PF-3758309**.

Baseline whole-genome transcriptome profiles of the five most sensitive (IC_50_ = 0.015 μmol/L; SW620, SW480, HCT116, RKO, and Colo205) and five most resistant (IC_50_ > 1 μmol/L; HCT15, LS1034, NCI-H508, SW1463, and GEO) CRC cell lines were obtained by microarray. By comparing the gene expression profiles between the PF-3758309-sensitive and -resistant cell lines, a clear pattern of differentially expressed genes was observed. Using significance analysis of microarrays with a stringent false discovery rate (FDR <0.001), genes associated with the epithelial or mesenchymal phenotype including E-cadherin, claudin-2/3, vimentin, or Zeb-1, were identified as down or up-regulated in the sensitive cell lines, respectively. Thus, an interesting finding from the global gene expression analysis was that genes associated with the EMT, were correlated with PF-3758309 sensitivity. We next compiled a list of genes known to be involved in EMT and generated a heat map of the five most sensitive or five most resistant CRC cell lines (**Figure 4**). CRC cell lines sensitive to PF-3758309 were associated with overexpression of mesenchymal (M) genes while resistant cell lines were enriched in epithelial (E) genes. The differential expressions in these genes were confirmed by RT-PCR (data not shown). We hypothesized that this EMT signature was associated with sensitivity to PF-3758309.

### PHENOTYPIC EFFECTS OF PF-3758309 ON ANCHORAGE-INDEPENDENT GROWTH, THREE-DIMENSIONAL TUMOR SPHEROID FORMATION, AND MIGRATION

**FIGURE 5:**
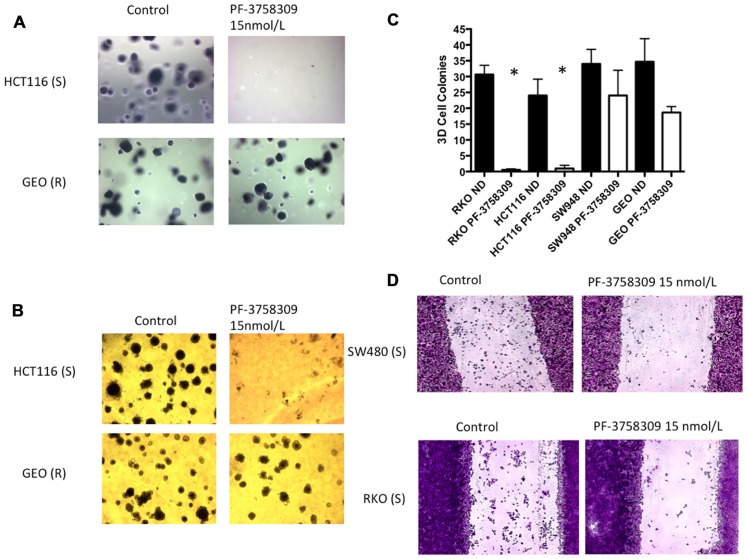
**Effects of PF-3758309 on anchorage-independent growth, spheroid formation and migration**. **(A)** HCT116 and GEO were plated in a soft agar assay untreated or treated with 15 nmol/L of PF-3758309 for 14 days. **(B)** HCT116 and GEO were plated in 3D culture in matrigel to observe spheroid formation. **(C)** Quantification of spheroid formation of two sensitive and two resistant cell lines. The average spheroids from three separate experiments are displayed. Data is presented as mean ± SEM (*t*-test: *p <0.001). **(D)** RKO and SW480 were evaluated for their migratory phenotype in the presence of PF-3758309. Cells grown to 80% confluence were cleared with a scratch and migration was measured by the ability of cells to migrate into scratch after 48 h.

Since the PAKs play an important role in Ras-driven anchorage-independent growth, 3D morphogenesis, and migration (Callow et al., 2002; O’Brien et al., 2006; Marlin et al., 2009), we next assessed the effects of PF-3758309 against 2-S (HCT116, RKO) and 2-R (GEO, SW948) CRC cell lines utilizing a soft agar clonogenic assay, 3D culture, and a scratch assay. As depicted in **Figure 5A**, PF-3758309 inhibited anchorage-independent growth in the sensitive but not the resistant CRC cell lines, and this was recapitulated in a 3D tumor spheroid assay (**Figures 5B,C**). Likewise, PF-3758309 inhibited the migration of two sensitive CRC cell lines as assessed using a scratch assay (the CRC cell lines resistant to PF-3758309 were not able to be assessed in this assay due to the lack of migratory activity in the absence of drug; **Figure 5D**).

### *IN VIVO* VALIDATION OF PF-3758309 SENSITIVITY AND ASSESSMENT OF EMT MARKERS

**FIGURE 6:**
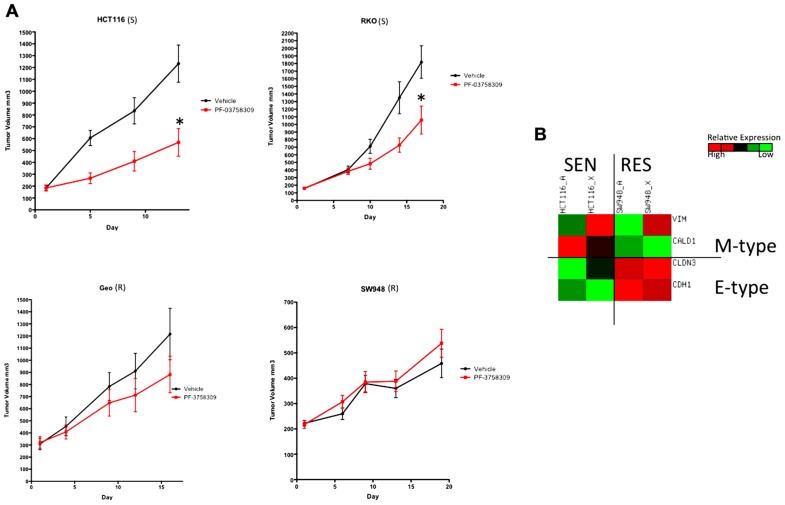
**Antiproliferative effects of PF-3758309 in cell line xenografts**. **(A)** Two sensitive and two resistant cell lines *in vitro* were implanted into athymic nude mice to confirm sensitivity *in vivo*. **(B)** Overall the EMT phenotype was recapitulated *in vivo*. HCT116_A and SW948_A are cell line gene expression. HCT116_X and SW948_X are gene expression from xenografted tumors.

To confirm the S or R phenotype of the cells lines *in vivo*, we used a nude mouse xenograft model of 2-S (HCT116 and RKO) and 2-R (SW948 and GEO) CRC cell lines and treated them with PF-3758309 for 14 days (**Figure 6A**). Tumor volumes were measured twice weekly throughout the experiment and the responses of the xenografts were consistent with the *in vitro* cell line results, i.e., HCT116 and RKO CRC cell lines exhibited a statistically significant tumor growth inhibition (*p* = 0.009 and 0.03, respectively) compared to vehicle, and responsiveness was associated with greater expression of the mesenchymal-associated genes, vimentin and caldesmon (**Figure 6B**).

### shRNA KNOCKDOWN OF EMT GENES *IN VITRO*

**FIGURE 7:**
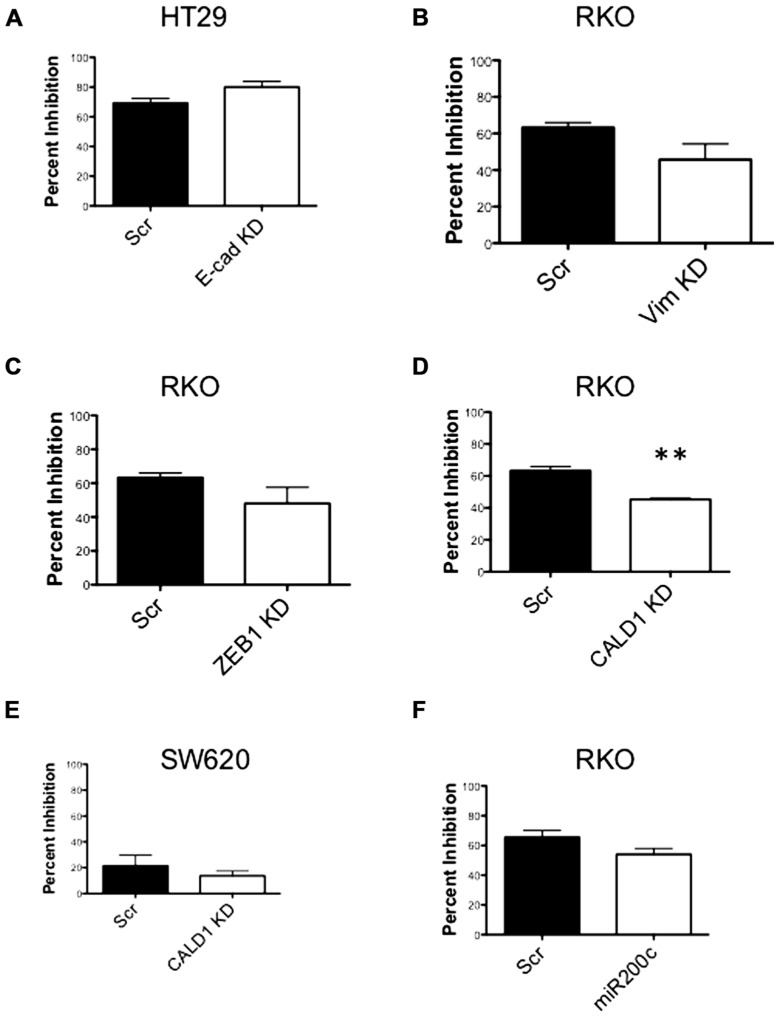
**Proliferation effects of knockdown of EMT-related genes**. **(A–E)** Transfected cell lines with EMT-related genes knocked down exposed to varying concentrations of PF-3758309. Proliferation by SRB (at 0.03 nmol/L of PF-3758309). **(F)** RKO cell line transfected with an miRNA 200c expression vector. Proliferation by SRB (at 0.03 nmol/L of PF-3758309).

**FIGURE 8:**
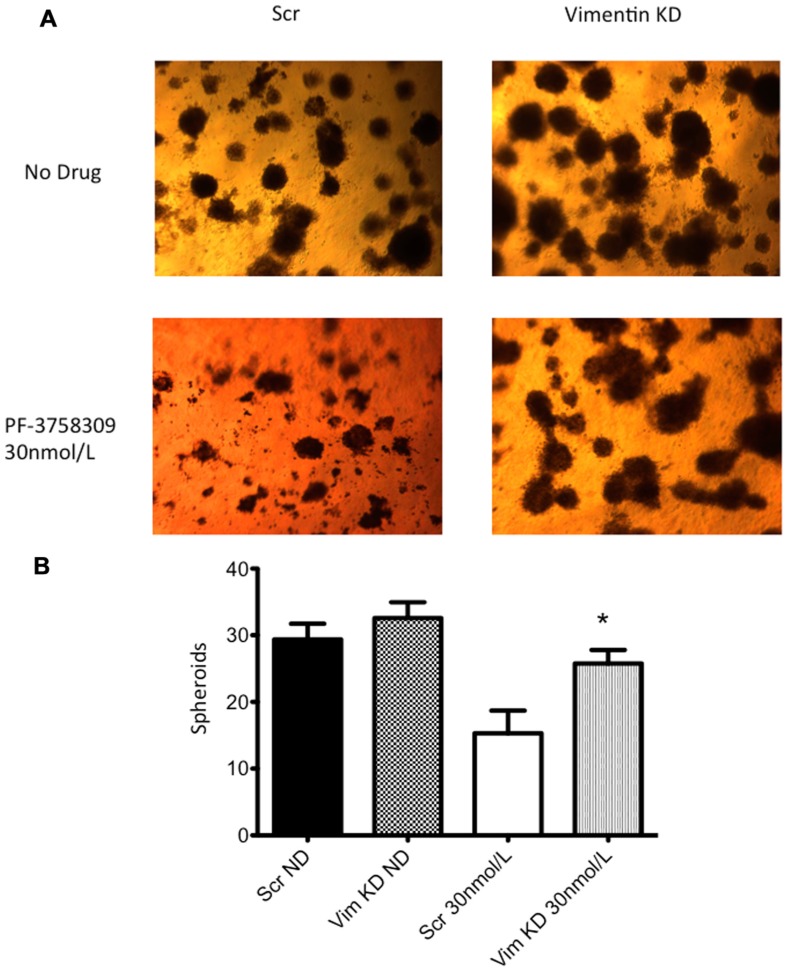
**Three-dimensional (3D) culture of RKO vimentin knockdown**. **(A)** The transfected cell lines were plated in 3D culture in matrigel to observe spheroid formation and treated with 30 nmol/L of PF-3758309 for 21 days. **(B)** The number of spheroids was counted from three independent experiments. Data presented as mean +/- SEM (t-test: *p <0.05, when compared to scramble control).

**FIGURE 9:**
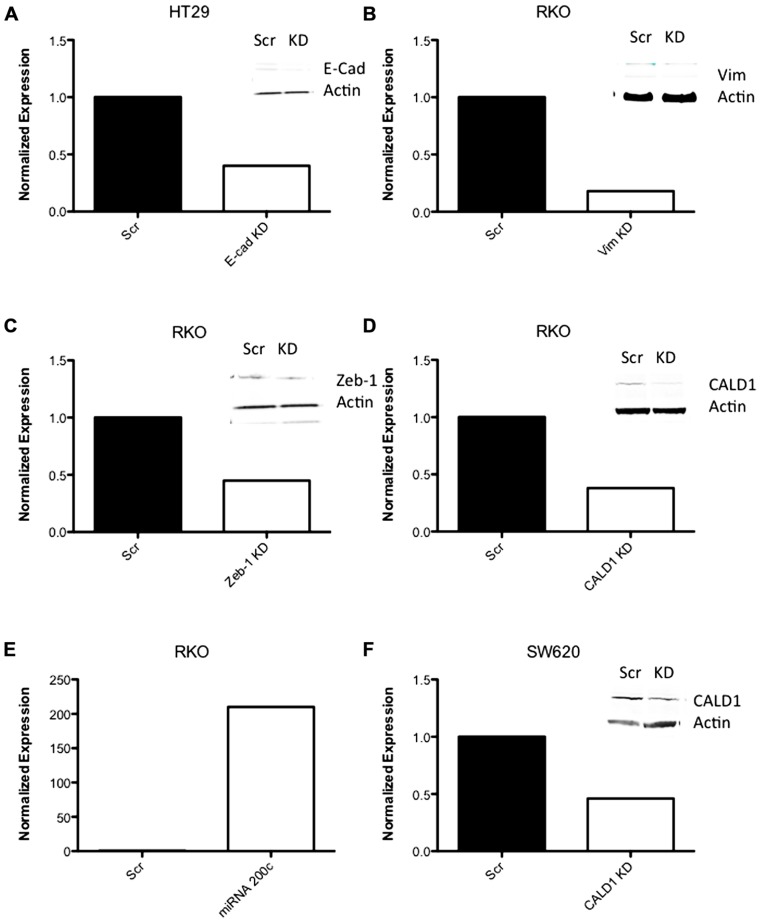
** RT-PCR data of shRNA knockdown and expression**. **(A–D, F)** RT-PCR data of CRC cell lines following transfection with shRNA to EMT-related genes. **(E)** RT-PCR of miRNA 200c following transfection with an expression vector containing miRNA 200c.

Next, to determine the functional role of EMT-associated genes in mediating responsiveness to PF-3758309, we performed shRNA knockdown and miRNA overexpression experiments. The PF-3758309 resistant CRC cell line, HT29, was transfected with shRNA for E-cadherin, whereas the PF-3758309 sensitive CRC cell line, RKO, was transfected with shRNAs against vimentin, caldesmon, Zeb-1, or the miRNA 200c expression vector (miRNA 200c suppresses Zeb-1 expression). These cell lines were chosen based on their ability to transduce the plasmid constructs efficiently. The hypothesis was that we would observe a shift toward sensitivity with the E-cadherin shRNA construct in the resistant cell line, while the other constructs would display a shift toward resistance in the sensitive cell line. The phenotype was analyzed by exposing the CRC cell lines to increasing concentrations of PF-3758309 and assessing proliferation. As hypothesized, in the resistant HT29 cells, shRNA knockdown of E-cadherin was associated with an increase in the antiproliferative effects toward a more sensitive phenotype, whereas shRNA knockdown of vimentin, caldesmon, and Zeb-1, or transfection with miRNA 200c resulted in a decrease in the antiproliferative effects toward a more resistant phenotype in the sensitive RKO cells (**Figures 7A–E**). Although the only statistically significant shift was observed in the RKO, CALD1 KD (*p* <0.01). A sensitive cell line, SW620 transfected with caldesmon shRNA also demonstrated a shift toward a more resistant phenotype (**Figure 7F**), although this was not significant. Next, to determine whether the shift toward resistance obtained *in vitro* could be recapitulated in a 3D tumor spheroid assay, the sensitive RKO cells containing a vimentin knockdown shRNA construct were exposed to PF-3758309 and scored in a 3D assay (**Figure 8**). As depicted in **Figure 8B**, there was a statistically significant increase (*p* <0.05) in the number of spheroids in the transfected cell line versus the scrambled control, consistent with the *in vitro* proliferation data. All shRNA and miRNA constructs were confirmed by RT-PCR (**Figure 9**).

### FUNCTIONAL CONSEQUENCES OF MODULATION OF EMT GENES *IN VIVO*

**FIGURE 10:**
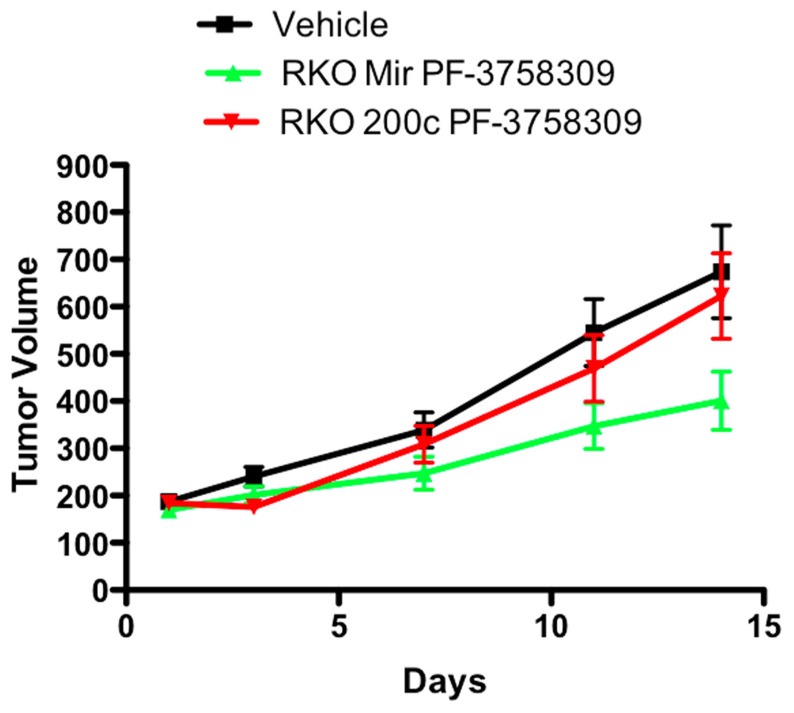
**Antiproliferative effects of an miRNA 200c transfected RKO cell line in a xenograft model**.

To determine whether the effect of modulating the EMT phenotype *in vitro* could be altered *in vivo*, the PF-3758309 sensitive RKO cell line was transfected with an miRNA 200c expression vector and implanted into immunodeficient mice. Our hypothesis was that miRNA 200c, by suppressing the mesenchymal phenotype-associated gene, Zeb-1, would result in less sensitivity of the RKO xenograft to PF-3758309 *in vivo*. As depicted in **Figure 10**, the RKO xenograft transfected with miRNA 200c exhibited a statistically significant shift (*p* <0.05) toward a more resistant phenotype when compared to the xenograft containing the empty vector control. No difference was observed in the growth kinetics of the xenograft containing the empty vector versus the xenograft with miRNA 200c in the vehicle group.

## DISCUSSION

p21-activated kinases are an attractive target for cancer therapy and clearly mediate pathways that drive the malignant phenotype (Ahn et al., 2011; Shrestha et al., 2011; He et al., 2012). PF-3758309 is a potent PAK inhibitor that demonstrates preclinical activity against a wide range of cancer cell types and has been investigated in a phase I clinical trial (Murray et al., 2010; Zhao and Manser, 2010). However, as with any first-in-class targeted therapy, defining mechanisms of resistance and/or responsiveness is necessary for effective clinical development. In the current study, we aimed to identify markers that were associated with responsiveness to the PAK inhibitor PF-3758309 in models of CRC. To accomplish this, we used *in vitro* models, gene expression analysis and molecular and pharmacologic tools *in vitro* and *in vivo*. In summary, our data suggest that CRC models exhibiting a mesenchymal phenotype are more sensitive to the PAK inhibitor, PF-3758309.

Interestingly in our panel of CRC cell lines we observed a broad range of proliferative sensitivity to PF-3758309, which was recapitulated, in other phenotypic assays such as anchorage-independent growth, 3D tumor spheroid formation and migration. Likewise, these effects were observed *in vivo* in cell line xenograft models. Similar results with this agent have been reported with other cancer types where *in vitro* data was confirmed *in vivo* (Murray et al., 2010). Interestingly, the wide range of responsiveness did not correlate with KRAS mutational status, PAK4 gene amplification, or alteration of PAK downstream effector molecules (**Figure 1** and data not shown). As previously reported (Murray et al., 2010), increases in apoptosis were observed that seemed to roughly correlate with sensitivity to PF-3758309.

One of the hallmarks of cancer is invasion and metastasis, which is hypothesized to occur by the loss of tumor epithelial morphology through a process known as EMT. During EMT, the primary tumor epithelial cells lose their cell–cell contact, acquire a more spindle shaped morphology and convert to a more motile and invasive phenotype (Thiery and Sleeman, 2006). Many molecular alterations occur during this process including loss of E-cadherin due to up-regulation of various transcription factors, including Zeb-1, Zeb-2, and Snail (Peinado et al., 2007). There is also increasing evidence that the miRNA 200 family can induce EMT by targeting Zeb-1, and Zeb-2. The EMT phenotype and drug resistance in cancer has been well established preclinically (McConkey et al., 2009; Chang et al., 2011; Jiao and Nan, 2012). For example, several studies have demonstrated that cells exhibiting a mesenchymal phenotype are more resistant to EGFR inhibitors and standard chemotherapy (Yauch et al., 2005; Yang et al., 2006; Frederick et al., 2007). Quite surprising was the finding in our studies that the epithelial phenotype conferred resistance to PF-3758309. One hypothesis is that CRC cell lines with an epithelial phenotype are less dependent on PAKs for proliferation and progression (Molli et al., 2009). Although the role of PAKs in EMT and cancer or how they relate to drug sensitivity/resistance has not been well described there have been reports of PAKs and EMT-like functions. Fort et al. (2011) describe a role of PAKs in cranial neural crest development, which is reminiscent of the early events of malignant progression with EMT properties. It is also known that PAKs can directly phosphorylate vimentin resulting in disassembly *in vitro* (Li et al., 2006) and it has been well documented that PAK4 regulates cell adhesion and anchorage-independent growth. Common to both PAK4 and mesenchymal-associated genes, is that when mutated or suppressed, anchorage-independent growth is inhibited (Qu et al., 2001; Callow et al., 2002). Although clearly this does not indicate a direct link between PAKs and EMT, one could hypothesize that malignant cells exhibiting a mesenchymal phenotype are more PAK-dependent and thus more sensitive to inhibition by PF-3758309 (Takeyama et al., 2010).

To explore the functional relationship between EMT markers and responsiveness to PF-3758309, we carried out several experiments targeting known genes and transcriptional regulators of EMT. We demonstrated that when mesenchymal-associated genes were suppressed in CRC cell lines exposed to PF-3758309, a shift to a more resistant phenotype was observed *in vitro* proliferation assays. This is in direct contrast to non-small cell lung cancer (NSCLC) cells, where the knock down of Zeb-1 results in both decreased proliferation and anchorage-independent growth in the presence of gefitinib, compared to parental control cells (Takeyama et al., 2010). Other studies have shown that gefitinib-resistant NSCLC cell lines regain sensitivity when E-cadherin is re-introduced (Witta et al., 2006). miRNA 200c, which suppresses Zeb-1, thereby inducing an epithelial phenotype, has been transfected into bladder, ovarian, and breast cancers, leading to restoration of chemosensitivity (Adam et al., 2009; Cochrane et al., 2009). In our studies, we hypothesized that the same type of experiment would lead to less responsiveness to PF-3758309, which indeed was observed *in vitro* and *in vivo*.

Recent reports have demonstrated that PAK1 and PAK4 can directly phosphorylate the S675 residue of β-catenin, which leads to stability and increase transcriptional activity (Zhu et al., 2011; Li et al., 2012). Clearly, these results are provocative and reinforce the need for further studies to delineate the relationships between PAKs/PAK inhibition to cancer stem cells and EMT.

In summary, PF-3758309 is a novel first-in-class inhibitor of the PAK family with selectivity toward PAK1 and PAK4. In our studies, we established that this novel agent demonstrates activity against preclinical CRC models cell lines with a range of molecular aberrations. Interestingly, we discovered that responsive models exhibit a more mesenchymal phenotype, indicating that rational combinations with epithelial subtype-directed agents such as cetuximab may result in more durable responses. PF-3758309 has completed phase I trials and further studies with this class of agents is ongoing.

## Conflict of Interest Statement

S. Gail Eckhardt – Research Funding, Fellowship Funding, and Honoraria from Pfizer. Erica L. Bradshaw-Pierce and BrionW.Murray are former and current employees of Pfizer, respectively.

## Footnotes

1 https://cabig.nci.nih.gov/

2 http://tanlab.ucdenver.edu/PAK4FrontiersARRAY.html
